# Identification of emotions evoked by music via spatial-temporal transformer in multi-channel EEG signals

**DOI:** 10.3389/fnins.2023.1188696

**Published:** 2023-07-06

**Authors:** Yanan Zhou, Jian Lian

**Affiliations:** ^1^School of Arts, Beijing Foreign Studies University, Beijing, China; ^2^School of Intelligence Engineering, Shandong Management University, Jinan, China

**Keywords:** human computer interface, emotion classification, deep learning, electroencephalographic, machine learning

## Abstract

**Introduction:**

Emotion plays a vital role in understanding activities and associations. Due to being non-invasive, many experts have employed EEG signals as a reliable technique for emotion recognition. Identifying emotions from multi-channel EEG signals is evolving into a crucial task for diagnosing emotional disorders in neuroscience. One challenge with automated emotion recognition in EEG signals is to extract and select the discriminating features to classify different emotions accurately.

**Methods:**

In this study, we proposed a novel Transformer model for identifying emotions from multi-channel EEG signals. Note that we directly fed the raw EEG signal into the proposed Transformer, which aims at eliminating the issues caused by the local receptive fields in the convolutional neural networks. The presented deep learning model consists of two separate channels to address the spatial and temporal information in the EEG signals, respectively.

**Results:**

In the experiments, we first collected the EEG recordings from 20 subjects during listening to music. Experimental results of the proposed approach for binary emotion classification (positive and negative) and ternary emotion classification (positive, negative, and neutral) indicated the accuracy of 97.3 and 97.1%, respectively. We conducted comparison experiments on the same dataset using the proposed method and state-of-the-art techniques. Moreover, we achieved a promising outcome in comparison with these approaches.

**Discussion:**

Due to the performance of the proposed approach, it can be a potentially valuable instrument for human-computer interface system.

## 1. Introduction

Emotion plays an essential role in the enjoyment of music, which consists of a large variety of affective states consistently reported by people while listening to music (Vuilleumier and Trost, 2015). Music is one of the crucial ways to express emotions. Different music can evoke various emotional responses from listeners. Listening to music is also an easy and effective way to change moods or reduce stress (Cui et al., 2022). In recent decades, music emotion recognition has become one hotspot in neuroscience. Plenty of music emotion recognition applications have achieved promising outcomes in different areas, including automated music composition, psychotherapy, and dancing generation with music (Eerola and Vuoskoski, 2012; Cui et al., 2022).

Electroencephalography (EEG) has received considerable attention in emotion state identification due to its simplicity, inexpensiveness, and portability (Alarcao and Fonseca, 2017). Specifically, EEG has been extensively employed in evaluating the impact of music on human brain activity, e.g., human emotion (Lin et al., 2006). For instance, Sammler et al. (2007) explored whether the valence of emotions would affect EEG power spectra and heart rate differently. The experiments collected pleasant and unpleasant emotions induced by a consonant and dissonant music using EEG signals. This study showed that pleasant music is significantly related to increased EEG theta power, while unpleasant music could significantly decrease heart rate. In the work of Balasubramanian et al. (2018), the authors analyzed the emotional responses of human beings to music in EEG. Ten healthy subjects (average age is 20) participated in this study. The self-assessment manikin (SAM) test assessed the subjects' perceived emotions when listening to music. The outcome from their experiments demonstrated an increase in the theta band of the frontal midline for liked music, and an increase in the beta band was found for disliked music. The publicly available dataset DEAP was presented in the work of Koelstra et al. (2011) for analyzing the human affective states. EEG and other physiological signals from 32 participants were recorded as each watched 40 1-min excerpts of music videos. Moreover, the participants must rate the videos regarding arousal, valence, like/dislike, dominance, and familiarity. In addition, Ozel et al. (2019) presented an approach for emotion recognition using time-frequency analysis of multivariate synchrosqueezing transform in multi-channel EEG signals. The evaluation of this study employed the DEAP, and a total of eight emotional states were considered by combining arousal, valence, and dominance. Lin et al. (2010) leveraged machine learning-based methods to classify EEG dynamics based on self-assessed emotional states when subjects listen to music. A framework was presented to optimize emotion recognition in EEG signals by extracting emotion-specific features from EEG recordings and enhancing the effectiveness of the classifiers. Zheng et al. (2017) aimed to identify EEG stability in the process of emotion recognition by comparing the performance of various feature extraction, feature selection, feature smoothing, and classification methods on the DEAP dataset and the SEED (Zheng and Lu, 2015) dataset. Generally, a regularized robust learning algorithm with differential entropy features yields the optimal outcome on the DEAP and SEED datasets. Using both a private dataset and the public dataset DEAP, Thammasan et al. (2017) investigated the influence of familiarity on brain activity in EEG signals. In the experiments, the subjects were asked to determine an equal number of familiar and unfamiliar songs; the datasets' outcomes both demonstrated the significance of self-emotion assessment according to the assumption that the emotional state is subjective when listening to music. Hou and Chen (2019) conducted experiments to extract the optimal EEG features induced by music evoking various emotions, including calm, joy, sadness, and anger. The 27-dimensional features were generated from EEG signals during the feature extraction process. A feature selection method was exploited to identify the features significantly related to the emotions evoked by music. Since music emotion recognition has attracted widespread attention with the enhancement of deep learning-based artificial intelligence applications, Han et al. (2022) surveyed music emotion recognition. The evaluation metrics, emotion recognition algorithms, datasets, and features involved were provided in detail. Recently, there have been numerous applications of deep learning methods in music classification and recognition (McIntosh, 2021; Eskine, 2022; Nag et al., 2022; Daly, 2023).

Several limitations still need to be resolved in the current automatic music emotion classification algorithms in EEG signals. On the one hand, for the machine learning-based methods, a set of optimal features is required to be extracted and selected from the EEG recordings. On the other hand, deep learning-based approaches, especially convolutional neural networks (CNNs), are prone to a need for global associations between long-range sampling signals due to the local receptive field issue of CNN-based models. Bearing the analysis mentioned above in mind, we proposed a spatial-temporal transformer-based pipeline for music emotion identification. The presented approach extracts the spatial features from EEG signals from different subjects listening to the same music. The temporal features are extracted from EEG signals from the same subject listening to the same type of music. Experimental results demonstrate that the performance of the proposed approach outperforms the state-of-the-art techniques in binary and ternary music emotion classification. The proposed approach could be a valuable instrument for various applications built upon music emotion recognition.

The remaining of this following content is organized as follows: First, the data samples used in this study and the details of the proposed transformer model are described in Section 2; Section 3 gives the experimental results of the proposed approach on the collected EEG recordings and the comparison between the state-of-the-art techniques and our proposed method; the discussion of the experimental results and the proposed method are given in Section 4; Finally, the conclusion of this study is given in Section 5.

## 2. Materials and methods

In this section, the process of collecting the EEG recordings used in this study is introduced first. Then, the content of the proposed deep learning model is given in detail.

### 2.1. Dataset

**Figure 1:**
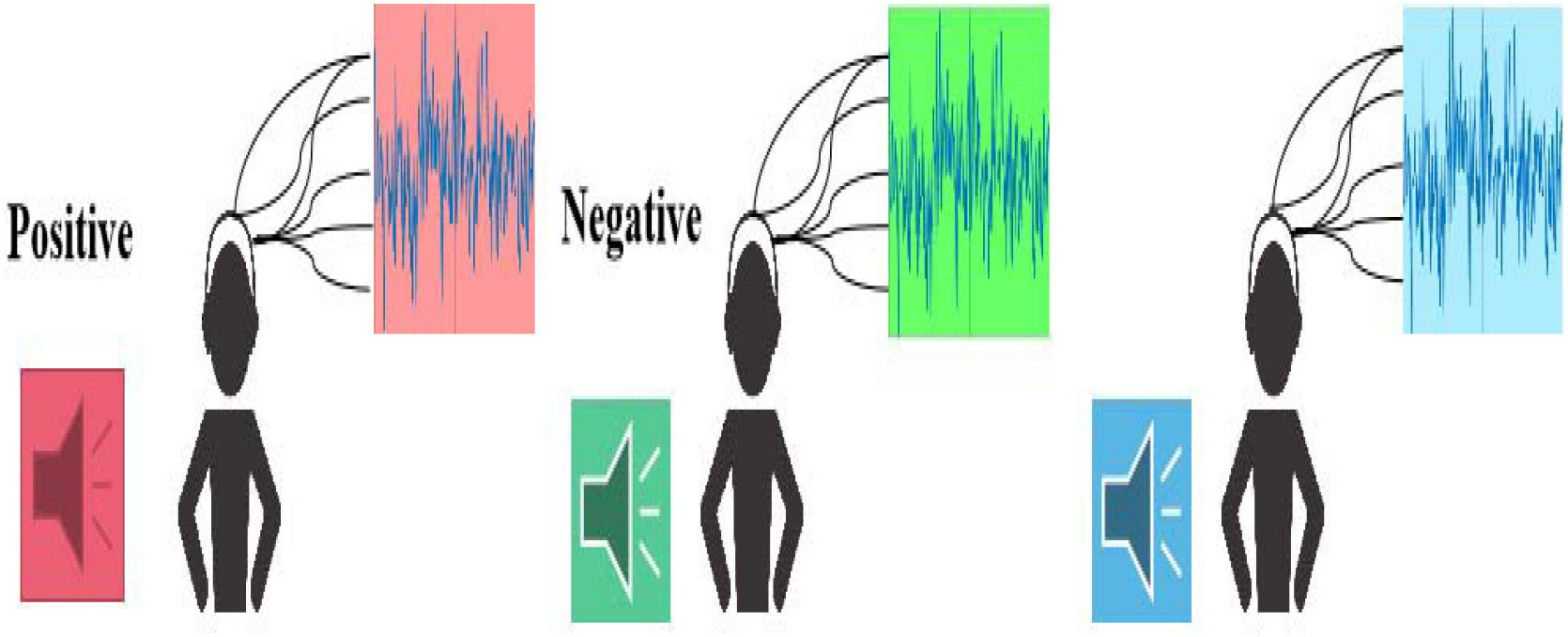
The workflow of the music emotion EEG signals collection.

A dataset was established by capturing three types of emotions, including positive, negative, and neural, from the EEG recordings. The entire workflow is illustrated in Figure 1.

Before the EEG collection process, the following preparations were performed:

- Ensure that the participants participated in this experiment on an entirely voluntary basis;
- Participants must read the experimental precautions and execution process, including the errors that could be caused by physical shaking and emotional tension;
- Participants need to fill in the personal information form and check whether the electrode is in good contact and whether the electrode cap is placed correctly;
- The participants put on the electrode cap, adjusted the best physical and psychological state, and pressed the button to start the test when ready.

During the EEG collection process, 32 participants (16 females and 16 males) aged 18–31 (mean age is 24.7) were enrolled, all working or studying at the same University. These 32 subjects are in good physical and mental condition, without mental illness or brain damage. In addition, two specialists (two females) at the elicitation of emotion majoring in psychology contributed to determine the adopted music clips.

During the data collection process, each subject needs to listen to 12 pieces of music excerpts (four pieces of positive music excerpts, four pieces of negative music excerpts, and four pieces of neutral music excerpts, as shown in Table 1), which are different kinds of music clips with a uniform duration of 1 min. To note that the sequence of playing the music clips is randomized for the participants to neutralize the retention effect of the emotion evoked by the previous music clip on the next one. The EEG acquisition equipment used is the Biosemi ActiveTwo system, with 32 EEG signal channels, using 512 Hz sampling, 128 Hz complex sampling (pre-processing), and using the 10-20 system EEG signals.

**Table 1:** The types and descriptions of the music excerpts used in this study.

**ID**	**Type**	**Title**	**Singer**
1	Positive	A man should strengthen himself	Zixiang Lin
2	Negative	A dream of misery	Wei Dou
3	Neutral	Reiki meditation	Not applicable
4	Positive	I really love you	Beyond
5	Negative	In case	SHIN
6	Neutral	Calm tech	Not applicable
7	Positive	Flying higher	Feng Wang
8	Negative	Collapse	Catcher in the Rye
9	Neutral	Let the sun shine	Not applicable
10	Positive	Friend	Huajian Zhou
11	Negative	Negative	Bill Do
12	Neutral	Make it rain	Not applicable

The positions of 32 EEG channels are selected according to the international 10-20 system, namely Fp1, AF3, F3, F7, FC5, FC1, C3, T7, CP5, CP1, P3, P7, PO3, O1, Oz, Pz, Fp2, AF4, Fz, F4, F8, FC6, FC, Cz, C4, T8, Cp6, Cp2, P4, P8, PO4, O2. The position distribution of electrode placement covers the four primary areas of the brain, with moderate spacing, which can effectively collect the required EEG raw data.

The specific process of EEG acquisition is shown in the following figure, mainly including the following steps:

- The Serial number of the progress of the experiment. Give the serial number through the prompt sound to let participants know what music is currently in progress;
- Collection of benchmark records. This process will last for 5 s. At this time, participants try to keep calm and record the mark of the beginning of the EEG signal;
- Music playback. This process will last for 75 s. The 15 s is the time for each music switch, and the 60 s is the time for the music to play. During this process, participants need to keep their body balance and reduce movement as much as possible;
- Self-assessment scoring. After listening to each music excerpt, the participants must evaluate themselves in time (−1 denotes negative, 0 denotes neutral, and +1 denotes positive). This process will last < 15 s, equal to the period for the music switch. In addition, the participants need to take a short rest after self-assessment according to their real emotional experience after listening to music;
- Start playing the next piece of music. Repeat the above two steps until all 12 music materials have been played.

The experiment uses the software provided by the company to play music. The CPU of the computer playing video is Core i3; the memory is 4 GB, and the Windows 10 operating system. Notably, the EEG signals were recorded while listening to music. All EEG signals were recorded between 9 and 11 a.m., and 2 and 5 p.m. to ensure the participants were not tired. Also, to avoid the noise from different sources, including Electro-Oculogram (EOG) and Electrocardiograph (ECG), all the subjects were instructed to keep their eyes closed during the EEG recording process.

For the binary classification task (positive and negative), 8 min (positive = 240 s and negative = 240 s) of sampled EEG signals were used for two types of emotions. Furthermore, the EEG samples from each electrode were divided into overlapped 240 epochs, each lasting 4 s. On the other hand, for the ternary classification task (positive, negative, and neutral), 12 min (positive = 240 seconds, negative = 240 s, and neutral = 240 s) of sampled EEG signals were used for three types of emotions. The EEG signals from each electrode were divided into overlapped 360 epochs, each lasting 4 s. Considering the balance of classification, the number of samples for each type of emotion used in both binary and ternary classification equals each other. In addition, the overlapped epochs were leveraged to decrease the possibility of over-fitting during the classification processes. Note that the complex sampling is 128 Hz for the EEG signals in this study. Thus each epoch of 4 s contains 512 sampling points in total.

### 2.2. Spatial-temporal transformer

**Figure 2:**
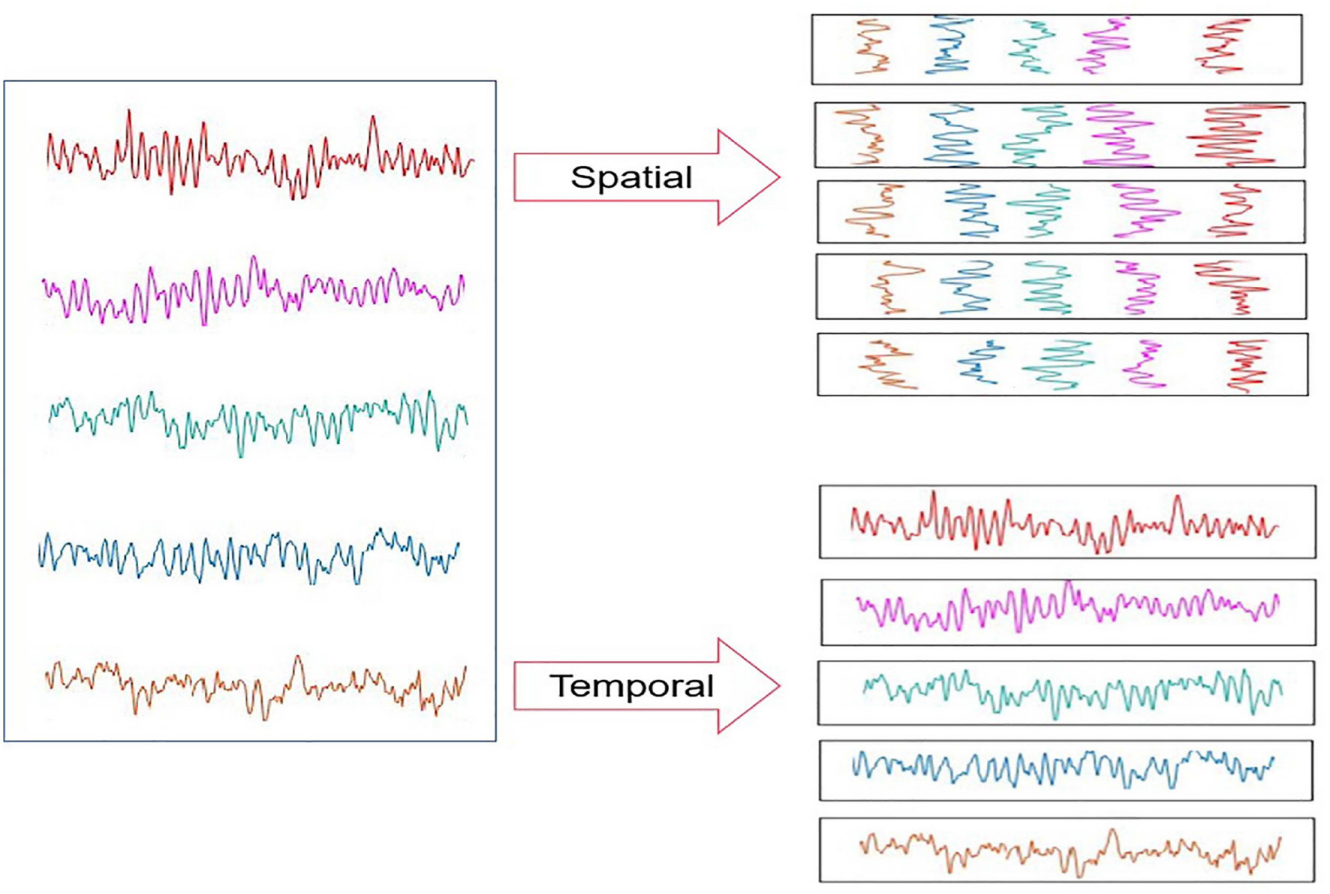
The input of the proposed spatial-temporal transformer.

In this section, details about the proposed spatial-temporal transformer model are provided. Note that this transformer is constructed using a self-attention mechanism, which has been extensively employed in many previous works (Fan et al., 2021; Liu et al., 2021; Wang et al., 2021). In addition, we conceived that the transformer-based deep learning architecture could produce competitive outcomes over the CNN-based algorithms, which are constrained by the issues caused by the local receptive field. The pipeline of the presented spatial-temporal transformer is shown in Figure 2, inspired by the model of ViT (Dosovitskiy et al., 2020) that leverages both the image patches and the corresponding sequence of these image patches as the input for the model.

In general, the input for the presented spatial-temporal transformer is composed of both the spatial and temporal epochs of the EEG signals captured from the same subject. Furthermore, to integrate the spatial and temporal associations between a batch of EEG signals (each group has eight epochs), the corresponding position embedding was fed into the proposed transformer (as shown in Figure 2). It is notable that each input batch of EEG epochs belongs to the same type of emotion.

**Figure 3:**
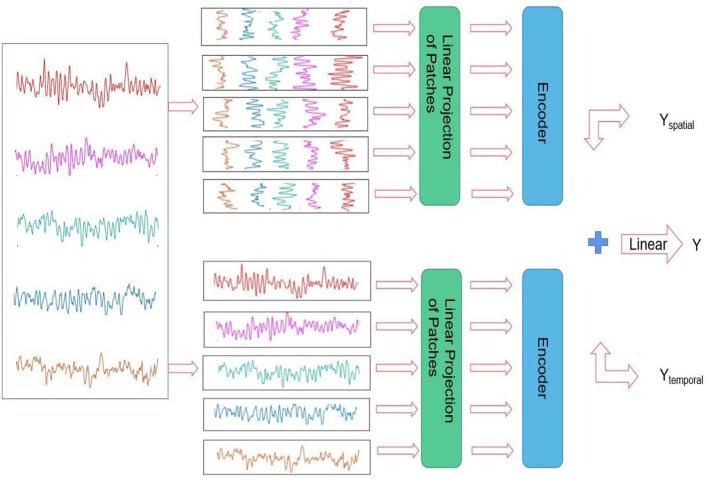
Architecture of the presented spatial-temporal transformer.

Suppose that H and W denote the height and width of the input of each channel in the proposed model for the EEG epochs divided from the original EEG signals, H = 5 and W = 512; Let C represent the number of channels, and the epochs were flattened and mapped into the vector in a length of D. Inspired by the embedding used in ViT (Dosovitskiy et al., 2020), we also added a learnable embedding along with the epoch embedding sequence. The output corresponding to the input epochs from the spatial channel is Yspatial, the output corresponding to the input epochs from the temporal channel is Ytemporal, and the composite output is denoted as Y (as shown in Figure 3).

**Figure 4:**
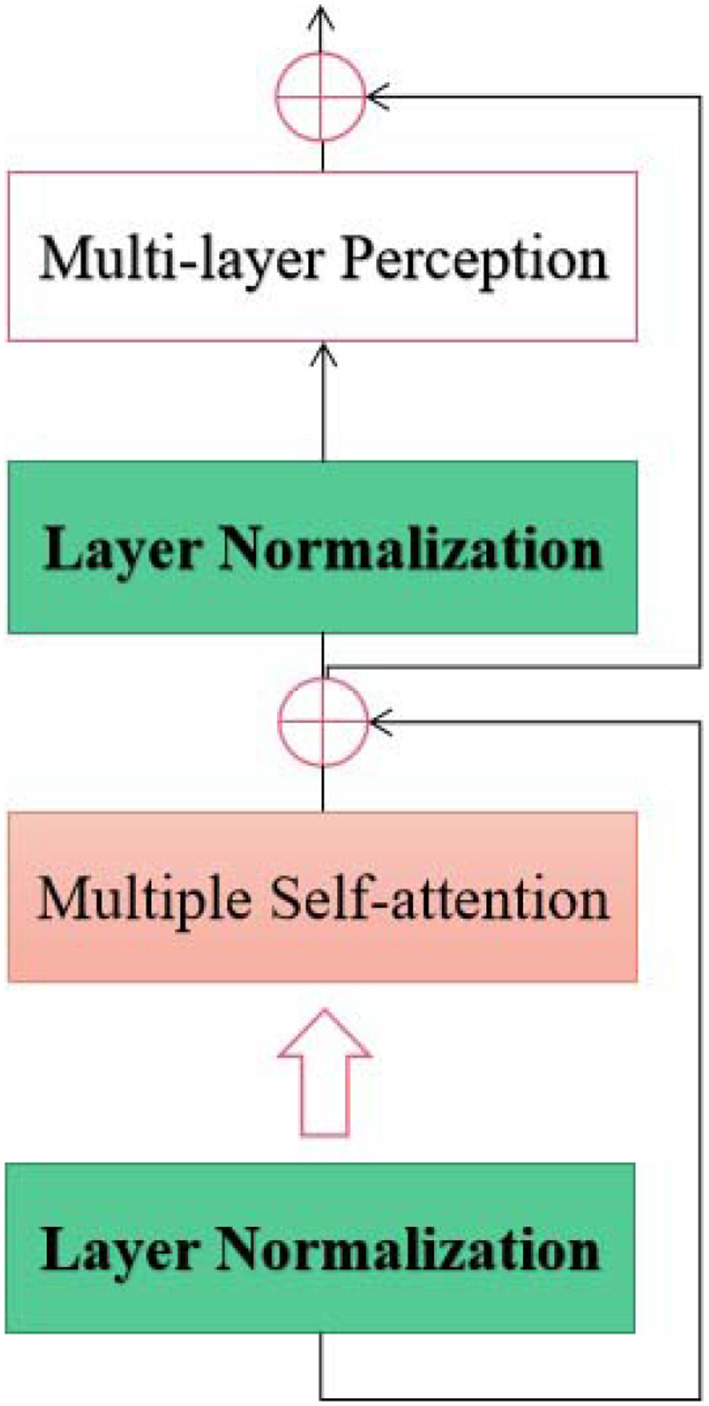
The Encoder Unit used in the proposed spatial-temporal transformer.

As shown in Figure 3, the proposed spatial-temporal transformer model consists of two channels designed for the spatial and temporal EEG epochs without sharing weighting parameters between the channels. According to the upper and bottom components on the right side of Figure 3, the upper and bottom channels are supposed to address the spatial and temporal information within the input EEG samples, respectively. Furthermore, inspired by the structure of ViT (Dosovitskiy et al., 2020) and the original transformer model (Vaswani et al., 2017), the proposed model adopts a sequence of token embedding and the component of the transformer Encoder (as shown in Figure 4).

The Encoder in each channel of the proposed transformer consists of *L* layers. Furthermore, each layer contains multiple self-attention units and a multi-layer perception unit. Along with the attention unit, the layer Normalization mechanism is exploited before both units. The multiple attention unit with *H* heads in the proposed model is constructed using the self-attention mechanism, which can be used to measure the similarity between each query and the keys by allocating a weight for each value (Vaswani et al., 2017). Thus, the output of the proposed approach can be obtained from a weighted sum of the outputs from the two channels. Moreover, a linear operator is leveraged to integrate the outputs from two channels, which can be mathematically formulated as (Equation 1):


(1) $$Y=Linear(layernNormalization(Y_{spatial},Y_{temporal}),$$

To be specific, besides the composite output *Y*, the outcomes of *Y*_*spatial*_ and *Y*_*temporal*_ can also be used to implement the classification of input EEG samples, respectively.

## 3. Results

In this section, the experimental results of the proposed method was provided for automated music emotion classification.

### 3.1. Implementation details

The proposed algorithm was realized using the PyTorch (Paszke et al., 2019) framework and two NVidia Telsa V100 Graphical Processing Units (GPUs) with 32GB RAM. The hyper-parameters for the proposed network were determined by using a trial-and-error fashion. To be specific, the model training was performed by adopting GELU (Hendrycks and Gimpel, 2016) activation function and AMSGrad optimizer (Reddi et al., 2019) with the learning rate of 0.001.

**Figure 5:**
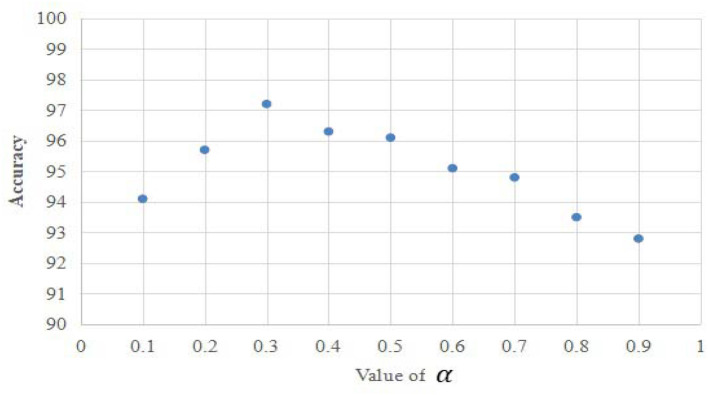
The optimal value of the hyperparameter α used in the experiments.

To evaluate the performance of the proposed approach, a set of experiments were carried out in sequence. To determine the optimal combination of the spatial and temporal channels in the proposed approach, we adopted the loss hyperparameter α and found the optimal value of α by training on half of the training samples before starting the following experiments (as shown in Figure 5). As shown in Figure 5, the optimal value of α is set to **0.3** in the experiments. Firstly, we carried out the ablation study by comparing the outcomes of the separate spatial channel, separate temporal channel, and the composite channels with each other. In addition, we evaluated the impact of number of heads and layers (*L*) of the proposed transformer model in the ablation study. We then conducted the experiments on all subjects in the manually collected dataset to build the baseline for the remaining experiments. In addition, the comparison experiments were conducted between the state-of-the-art deep learning methods and ours on the manually collected dataset. Finally, we provided the comparison between the state-of-the-art algorithms and ours on the publicly available DEAP dataset (Koelstra et al., 2011). To be specific, sensitivity, specificity, and accuracy were leveraged as the evaluation metrics in the experiments.

Meanwhile, to improve the performance of the proposed method, an integrated loss function was used by integrating both the spatial and temporal components, as shown in Equation (2).


(2) $$\begin{array}{l} Loss=\alpha Loss_{spatial}+\left(1-\alpha \right)Loss_{temporal}, \end{array}$$

where α denotes the weighting parameter for determining the combination of spatial and t temporal channels of the proposed model, *Loss*_*spatial*_ and *Loss*_*temporal*_ represent the cross entropy loss of the separate channels in the proposed transformer, respectively. In addition, the following metrics were used to evaluate the performance of the techniques in the experiments, including sensitivity, specificity, and accuracy, which are provided in Equations (3)–(5).


(3) $$\begin{array}{l} Sensitivity=\frac{TP}{TP+FN}, \end{array}$$

(4) $$\begin{array}{l} Specificity=\frac{TN}{TN+FP}, \end{array}$$

(5) $$\begin{array}{l} Accuracy=\frac{TP+TN}{TP+FN+TN+FP}, \end{array}$$

where TP, FN, TN, FP denote true positive, false negative, true negative, and false positive, respectively.

Furthermore, a 10-fold cross-validation mechanism was employed in the experimental process. First, all the input EEG samples were divided into ten subsets with the same number of samples. In each round of ten rounds, one subgroup was taken as the testing set, and the remaining groups were used as the training set. At last, the average of 10-folds was leveraged as the outcome for the methods used in the experiments.

### 3.2. Ablation study

#### 3.2.1. Hybrid model and individual transformers

**Figure 6:**
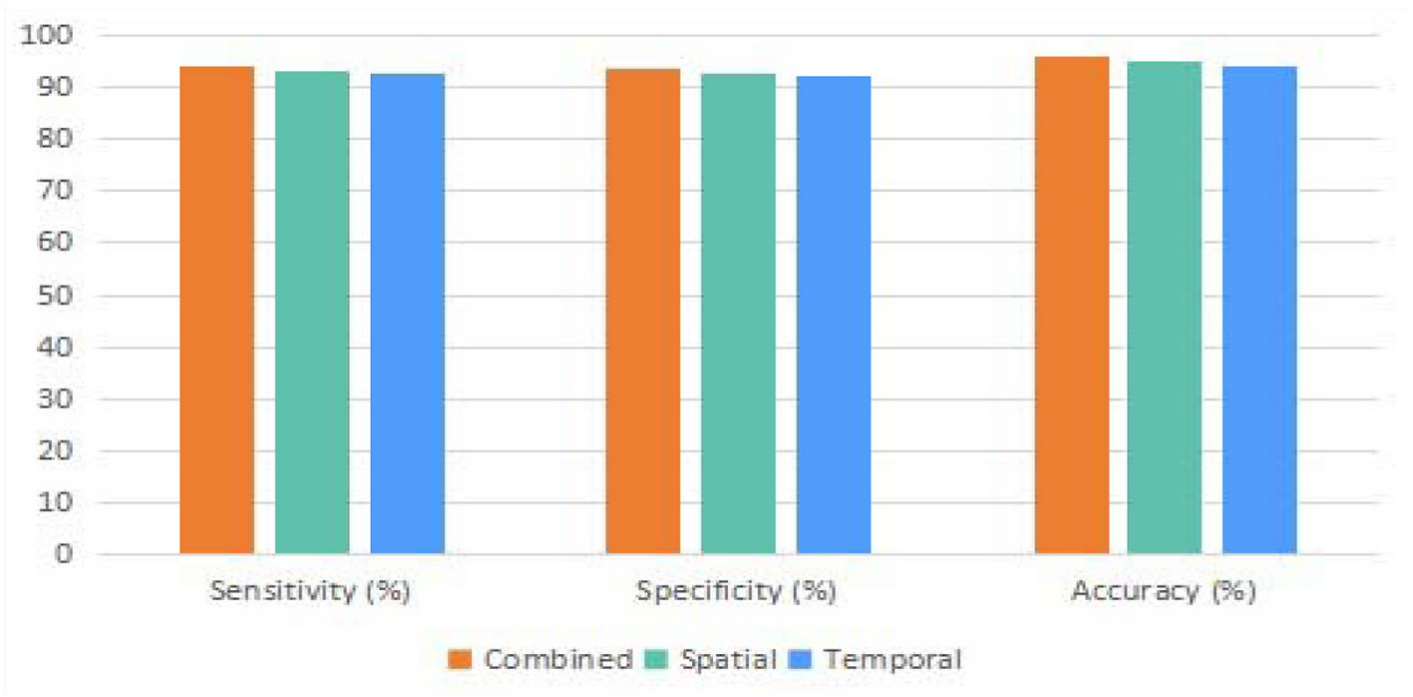
The binary classification comparison between the combined, spatial, and temporal versions of the proposed method.

The proposed approach could be treated as a hybrid model due to the spatial and temporal channels employed. Therefore, we first compared the proposed model with individual channels and the model with both channels. Due to the difference in sensitivity, specificity, and accuracy values of the three different models, including spatial, temporal, and combined models in the binary classification task, it can be observed that the integration of the two channels is superior in various performances over the individual channels (as shown in Figure 6).

#### 3.2.2. Impact of number of heads and layers of the proposed transformer

In addition, with the proposed hybrid model with both spatial and temporal channels, we further evaluated the impact of number of heads and layers on the binary-classification performance of the proposed the transformer model. As shown in Table 2, the optimal combination of the number of heads and layers is 4 and 8.

**Table 2:** The influence of number of heads and number of layers on the binary-classification outcome of the proposed model.

**Model**	**Number of heads (H)**	**Number of layers (L)**	**Accuracy (%)**
STT_1_4	1	4	96.1
STT_1_8	1	8	96.7
STT_1_12	1	12	96.6
STT_2_4	2	4	95.8
STT_2_8	2	8	96.7
STT_2_12	2	12	97.1
STT_4_4	4	4	96.8
STT_4_8	4	8	**97.3**
STT_4_12	4	12	97.1

The bold values denote the best performance.

To ensure that the number of weighting parameters of the proposed model remains at a low level, we did not take more combinations of the number of heads and layers (e.g., 12 or 24) in the ablation study.

### 3.3. Binary and ternary classification outcomes of the proposed approach

**Figure 7:**
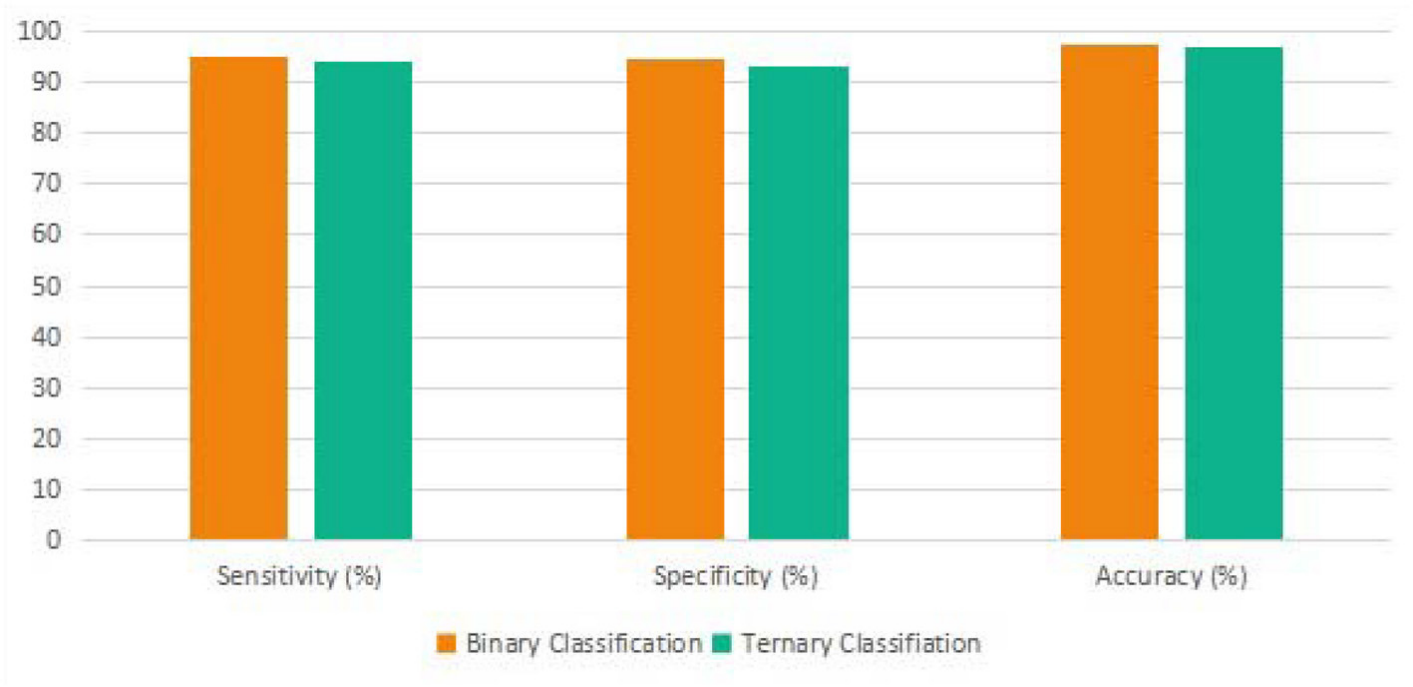
The binary and ternary classification outcome of the proposed transformer.

As shown in Figure 7, the sensitivity, specificity, and accuracy of the proposed approach in the binary classification task are 95.3, 94.8, and 97.3%. Meanwhile, the sensitivity, specificity, and accuracy of the proposed approach in the ternary classification task are 94.1, 93.2, and 97.1%. Note that both the evaluation metrics generated from the binary classification are more significant than the ternary classification by using the proposed algorithm since there are more samples and more types of samples in the ternary classification process.

### 3.4. Comparison experiments between the state-of-the-arts and the proposed approach

Furthermore, the following algorithms were incorporated into the comparison experiments between the state-of-the-art techniques and the presented approach, including U-Net (Ronneberger et al., 2015), Mask R-CNN (He et al., 2017), ExtremeNet (Zhou et al., 2019), TensorMask (Chen et al., 2019), Visual Transformer (Wu et al., 2020), ViT (Dosovitskiy et al., 2020), MViT (Fan et al., 2021), PVT (Wang et al., 2021), PiT (Heo et al., 2021), and Swin Transformer (Liu et al., 2021). As shown in Tables 3, 4, the proposed approach has achieved superior performance over the state-of-the-art algorithms.

**Table 3:** Binary classification comparison between the state-of-the-arts and the proposed approach.

**Method**	**Sensitivity (%)**	**Specificity (%)**	**Accuracy (%)**
U-Net (Ronneberger et al., 2015)	82.3	83.1	84.8
Mask R-CNN (He et al., 2017)	82.5	82.9	83.6
ExtremeNet (Zhou et al., 2019)	81.9	84.7	85.1
TensorMask (Chen et al., 2019)	82.9	83.8	84.7
Visual Transformer (Wu et al., 2020)	85.5	84.1	86.2
ViT (Dosovitskiy et al., 2020)	86.7	87.1	88.5
MViT (Fan et al., 2021)	89.4	87.9	90.3
PVT (Wang et al., 2021)	89.7	86.5	90.1
PiT (Heo et al., 2021)	92.4	90.5	93.8
Swin Transformer (Liu et al., 2021)	91.7	92.5	94.4
Pan and Zheng (2021)	91.2	90.8	90.4
Shen et al. (2020)	93.6	93.2	93.9
Kan et al. (2022)	94.2	93.9	94.3
Rudakov (2021)	95.2	94.5	95.8
The proposed approach	**95.3**	**94.8**	**97.3**

The bold values denote the best performance.

**Table 4:** Ternary classification comparison between the state-of-the-arts and the proposed approach.

**Method**	**Sensitivity (%)**	**Specificity (%)**	**Accuracy (%)**
U-Net (Ronneberger et al., 2015)	80.5	81.6	82.2
Mask R-CNN (He et al., 2017)	81.7	82.1	82.8
ExtremeNet (Zhou et al., 2019)	81.2	83.1	84.3
TensorMask (Chen et al., 2019)	82.3	83.1	85.2
Visual transformer (Wu et al., 2020)	83.4	84.2	85.9
ViT (Dosovitskiy et al., 2020)	85.6	86.9	88.7
MViT (Fan et al., 2021)	88.6	86.7	88.1
PVT (Wang et al., 2021)	88.2	87.2	89.5
PiT (Heo et al., 2021)	90.2	89.4	91.1
Swin transformer (Liu et al., 2021)	91.2	91.9	92.5
Pan and Zheng (2021)	90.7	91.0	90.2
Shen et al. (2020)	92.8	92.3	92.9
Kan et al. (2022)	93.5	93.8	93.9
Rudakov (2021)	93.8	**93.5**	95.3
The proposed approach	**94.1**	93.2	**97.1**

The bold values denote the best performance.

To note that the specificity of the algorithm (Rudakov, 2021) is superior over the proposed approach.

To evaluate the performance of the proposed approach in a fair fashion, we further evaluated the performance of the proposed approach and the state-of-the-art algorithms (Shawky et al., 2018; Yang et al., 2018, 2019; Xing et al., 2019; Shen et al., 2020; Pan and Zheng, 2021; Rudakov, 2021; Kan et al., 2022; Zhang et al., 2022) on the DEAP dataset (as shown in Table 5). Accordingly, we used the DEAP dataset during the training phase of the proposed approach in this stage.

**Table 5:** Comparison between the state-of-the-arts and the proposed approach on DEAP dataset.

**Method**	**Detail**	**Accuracy**	
		**Valence**	**Arousal**
Xing et al. (2019)	LSTM^*^	81.10	74.38
Shawky et al. (2018)	CNN	87.44	88.49
Yang et al. (2019)	CNN	90.01	90.65
Pan and Zheng (2021)	CNN	90.26	88.90
Yang et al. (2018)	LSTM	90.82	86.13
Shen et al. (2020)	CRNN^**^	94.22	94.58
Zhang et al. (2022)	GAN^***^	93.52	94.21
Kan et al. (2022)	Contrastive Learning	94.72	92.65
Rudakov (2021)	CNN	96.28	96.62
Ours	Transformer	**96.36**	**96.91**

* LSTM denotes long short term memory.

** CRNN denotes convolutional recurrent neural network.

*** GAN denotes generative adversarial network.

The bold values denote the best performance.

## 4. Discussion

In this study, a dual-channel transformer for music emotion classification was presented. The proposed model consists of two channels designed to extract spatial and temporal information from the EEG signals. To our best knowledge, this is an early work of transformer architecture in this type of machine-learning task. From the experimental results, it can be observed that our method has achieved superior performance (Binary classification: sensitivity 94.3%, specificity 93.8%, and accuracy 96.8%; Ternary classification: sensitivity 93.6%, specificity 92.2%, and accuracy 95.3%) over the state-of-the-art algorithms in both the binary and ternary classification tasks. To note that the neutral emotions evoked by the music clips get more misclassified than both the positive and negative emotions in the experiments. The competing methods used in the comparison experiments include the CNN-based and transformer-based deep learning models, which focus on local receptive and global receptive fields during the classification tasks, respectively. Since the spatial and temporal information can be extracted from the input EEG signals, the proposed approach has achieved superior performance over both CNNs and transformers. Notably, the input of a batch of EEG signals could be treated the same as an image for the CNNs and vision transformers mentioned in the comparison experiments.

Furthermore, according to the results of the ablation study, the combination of the spatial and temporal channels in the proposed transformer network is superior to the individual spatial channel or temporal channel. Meanwhile, the outcome of the proposed model in the ablation study has also proved that a hybrid model could be a valuable mechanism for enhancing the performance of individual deep-learning models. In addition, the impact of number of heads and layers of the proposed transformer was evaluated in another ablation study. The corresponding results demonstrate that the optimal number of heads and layers of are 12 and 6 for the proposed model.

Notably, the proposed music emotion classification framework is inspired by the ViT (Dosovitskiy et al., 2020) architecture. Different from the original transformer (Vaswani et al., 2017) used for natural language processing and the vision transformers (He et al., 2017; Chen et al., 2019; Zhou et al., 2019; Dosovitskiy et al., 2020; Wu et al., 2020; Fan et al., 2021; Heo et al., 2021; Liu et al., 2021; Wang et al., 2021) designed for image analysis, our method is primarily exploited to classify the EEG recordings. Moreover, the proposed approach needs to adapt to the requirements of EEG classification by using both the spatial and temporal modules. Moreover, the self-attention mechanism first presented in the work of Vaswani et al. (2017), the association between the global associations between the EEG fragments could be fully exploited and unveiled. Moreover, this is a primary strategy to enhance the discriminating ability of the proposed transformer.

In addition, this study has some limitations despite its contributions. First, we leveraged a private dataset in the experiments, and the publicly available dataset should be considered in the following steps. Secondly, the weighting parameters used in the proposed approach were selected using a trial-and-error strategy, which might not be the optimal choice. Moreover, a strategy with more interpretability should be exploited instead. Furthermore, the training process of the proposed approach was time-consuming, which could be resolved using more powerful GPU platforms. However, to implement the proposed approach in practical applications, the computation resources and the execution time should be decreased through lightweight parameter configuration. In the future, more types of emotions in music will be incorporated into our study to satisfy the requirements of different categories of the practical applications.

## 5. Conclusion

In this study, the transformer-based deep learning model generally enhances the performance of music emotion classification in EEG recordings compared with traditional deep learning techniques. Specifically, the ability of the global receptive field provided by the transformer is conceived as beneficial for unveiling the long-range associations between different components in EEG signals. Both the binary classification and ternary classification of emotions evoked by music of the proposed approach are promising. In addition, the proposed approach has achieved superior performance over the state-of-the-art deep learning techniques.

In the future, we will incorporate more participants and more music clips from various countries and cultures to improve the robustness of the proposed approach. In addition, more types of emotions evoked by music will also be taken into consideration in our future work.

## Data availability statement

The raw data supporting the conclusions of this article will be made available by the authors, without undue reservation.

## Ethics statement

The studies involving human participants were reviewed and approved by the Shandong Management University's Human Research Ethics Committee (2023031501). The patients/participants provided their written informed consent to participate in this study.

## Author contributions

JL: conceptualization, formal analysis, funding acquisition, methodology, project administration, and supervision. YZ and JL: data curation. YZ: investigation, validation, visualization, and writing—original draft. All authors contributed to the article and approved the submitted version.
